# Development of a Fluorophore-Bound l-Tryptophan Derivative for Evaluating Indoleamine 2,3-Dioxygenase Activity by HPLC with Fluorescence Detection: An In Vivo Microdialysis Study Using Rat Kidney

**DOI:** 10.3390/molecules31020283

**Published:** 2026-01-13

**Authors:** Mayu Onozato, Reika Aoki, Mai Yamaguchi, Honoka Fujimoto, Tatsuya Sakamoto, Takeshi Fukushima

**Affiliations:** Faculty of Pharmaceutical Sciences, Toho University, 2-2-1 Miyama, Funabashi-shi 274-8510, Chiba, Japan; mayu.onozato@phar.toho-u.ac.jp (M.O.); 1019003a@st.toho-u.jp (R.A.); 1019217y@st.toho-u.jp (M.Y.); 1021202f@st.toho-u.jp (H.F.); tatsuya.sakamoto@phar.toho-u.ac.jp (T.S.)

**Keywords:** indoleamine 2,3-dioxygenase, microdialysis, fluorescence, HPLC, rat kidney

## Abstract

Evaluating the activity of indoleamine 2,3-dioxygenase (IDO), the rate-limiting enzyme in tryptophan (Trp) metabolism, is important because IDO is involved in immune tolerance and drives the production of Trp metabolites implicated in psychiatric disorders and cancer. This study aimed to design and develop a novel fluorescent l-Trp derivative to fluorometrically monitor Trp-catabolizing enzyme activity via IDO. To evaluate IDO activity in vivo, 7-*N*,*N*-dimethylamino-2,1,3-benzoxadiazole (DBD), a fluorophore, was covalently bound at the 5-position of the indole ring in Trp to produce 5-DBD-l-Trp. An in vivo microdialysis (MD) study was conducted using the kidneys of Sprague–Dawley rats. Specifically, 5.0 μM 5-DBD-l-Trp in phosphate-buffered Ringer’s solution was infused into the rats, and the MD sample was analyzed via high-performance liquid chromatography with fluorescence detection. In the MD sample, two fluorescence peaks other than 5-DBD-l-Trp were observed during the 5-DBD-l-Trp infusion, and the main metabolite peak was proposed to be 5-DBD-kynurenine, verified by liquid chromatography-tandem mass spectrometry. The intensity of the fluorescent peak was significantly attenuated by co-infusion with an IDO inhibitor, 1-methyl-d-Trp. These results suggest that 5-DBD-l-Trp may be metabolized by renal IDO and can be used to evaluate IDO activity in vivo.

## 1. Introduction

**Scheme 1:**
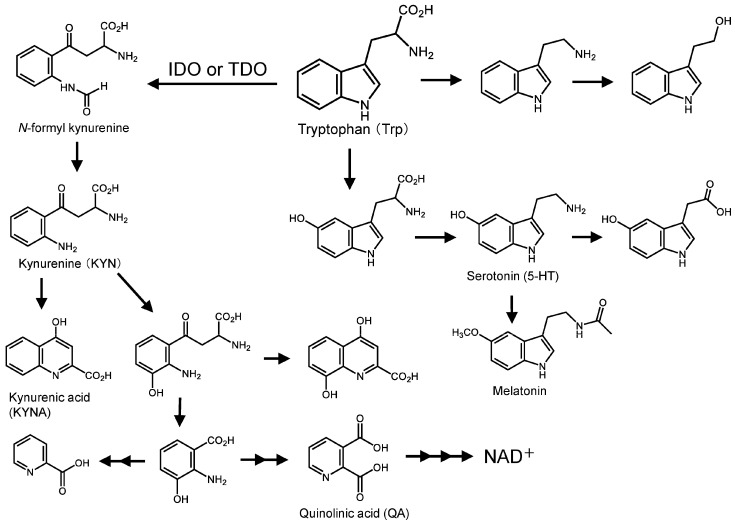
Tryptophan metabolism pathway.

One of the essential amino acids, l-tryptophan (Trp), is known to be metabolized into several bioactive metabolites, such as l-kynurenine (KYN), kynurenic acid (KYNA), picolinic acid (PA), serotonin, and related metabolites (Scheme 1). Trp is metabolized via two pathways: the kynurenine (KYN) pathway and the serotonin pathway. The KYN pathway is reportedly responsible for approximately 90% of Trp metabolism in mammals [1]. Two enzymes, indoleamine 2,3-dioxygenase (IDO) and tryptophan 2,3-dioxygenase (TDO), are the rate-limiting enzymes for Trp metabolism in the KYN pathway [1]. Abnormal regulation of the KYN pathway causes several diseases [2], and IDO expression is involved in immune tolerance and cancer therapeutics [3]. Therefore, selective inhibitors of IDO are expected to emerge as novel anti-cancer drug candidates [4].

Various determination methods have been used to analyze bioactive Trp metabolites, especially using chromatography techniques [5]. Recently, high-performance liquid chromatography–mass spectrometry (HPLC–MS) has become popular for the determination of trace Trp metabolites in biosamples [5]. By determining both Trp and KYN levels in tissue homogenates using this chromatographic technique, an estimation of Trp-catabolizing activity, specifically IDO or TDO activity, has been obtained by calculating the ratio of KYN to Trp levels [6,7,8,9].

However, to evaluate enzymatic activity in the tissue of experimental animals, in vivo microdialysis (MD) is a suitable technique [10]. MD is generally a sampling method of endogenous, particularly hydrophilic compounds such as amino acids and neurotransmitters, from the tissues of experimental animals under anesthesia, via a narrow probe embedding a permeable membrane with a constant flow of physiological perfusate. Using the MD technique, evaluation of enzyme activity in tissues is possible by adding an artificial fluorogenic substrate into the perfusate and measuring the fluorescence observed from the products [10]. Previously, we successfully evaluated the activity of renal d-amino acid oxidase, one of the susceptibility gene products for schizophrenia [11], in the kidneys of rats using renal MD experimentation [12]. Additionally, it is preferable to utilize high-performance liquid chromatography (HPLC) with fluorescence detection, as it is easier than liquid chromatography–mass spectrometry (LC–MS).

**Figure 1:**
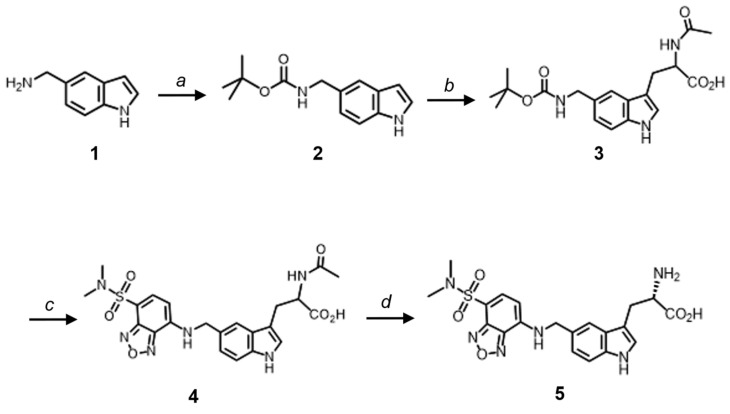
Preparation scheme of a fluorescent Trp derivative, 5-DBD-l-Trp. (*a*) Boc_2_O, Triethylamine in ethanol at 25 °C; (*b*) serine, DMAP, acetic acid, and acetic anhydride at 80 °C; (*c*) (1) trifluoroacetic acid at room temperature, (2) DBD-F, diisopropylethylamine, in ethanol at room temperature; (*d*) acylase I and cobalt (II) chloride in phosphate buffer (pH 7.5) at 45 °C. DBD, 4-*N*,*N*-dimethylamino-2,1,3-benzoxadiazole; DMAP, 4-(dimethylamino)pyridine.

In this study, we aimed to design and develop a novel fluorescent l-Trp derivative, which enabled us to fluorometrically monitor Trp-catabolizing enzyme activity via IDO or TDO (Figure 1). The fluorophore, 4-*N*,*N*-dimethylamino-2,1,3-benzoxadiazole (DBD), was chosen because its excitation and emission maxima occur at longer wavelengths than those of endogenous aromatic compounds, reducing spectral overlap and background interference. To date, DBD has been used for HPLC with fluorescence detection since the first report in 1989 [13,14]. The fluorescent Trp derivative, 5-DBD-l-Trp (Japanese Patent Application No. 2025-024126), was used to monitor Trp metabolism in an in vivo microdialysis (MD) study in Sprague–Dawley rat kidney, where both IDO and TDO are present [15]. The fluorescent 5-DBD-KYN, which corresponds to the l-Trp metabolite KYN, was detected in the MD samples from rat kidney by HPLC with fluorescence detection.

## 2. Results

### 2.1. Preparation of 5-DBD-l-Trp

The synthetic scheme of 5-DBD-l-Trp is presented in Figure 1. 5-DBD-l-Trp was synthesized from an indole derivative (**1**), as described in previous studies [16,17,18,19]. A primary amine with indole (**1**), employed as a starting material, was first protected by (Boc)_2_O to produce (**2**). Subsequently, an *N*-acetyl serine moiety, a racemate, was attached at the 3-position of the indole ring using a reaction described in previous studies [16,17,18,19] to produce (**3**).

After deprotection of the *tert*-butyloxy group, the fluorophore DBD was attached at the 5-position of Trp to produce (**4**) via a three-step reaction.

In the final synthetic process, the acetylamide bond of (**4**) was hydrolyzed by acylase I, which was used for the optical resolution of 5-DBD-Trp to prepare the optically active l-form of 5-DBD-Trp. Finally, the desired product was purified by flash column chromatography, yielding 5-DBD-l-Trp as an orange solid.

**Figure 2:**
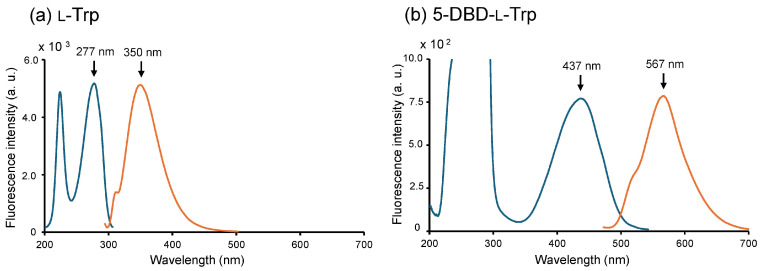
Excitation (blue) and fluorescence (red) spectra of l-Trp (**a**) (1.0 µM) and 5-DBD-l-Trp (**b**) (10 µM). All determinations were carried out using a spectrofluorometer, F-7000, and details are described in Section 4.3. DBD, 4-*N*,*N*-dimethylamino-2,1,3-benzoxadiazole.

Figure 2 shows the excitation and fluorescence spectra of l-Trp (a) and the prepared 5-DBD-l-Trp (b) dissolved in CH_3_CN. As shown in Figure 2b, fluorescence at 567 nm originating from 5-DBD-l-Trp was observed, whereas no fluorescence at 567 nm was observed for l-Trp (Figure 2a). These results indicate that the DBD moiety was successfully attached to Trp in the prepared 5-DBD-l-Trp, because the reported fluorescence wavelength of the DBD moiety is approximately 520–600 nm [13,14].

### 2.2. Results of In Vivo MD Study in Rats

#### 2.2.1. MD Study in Rat Kidneys

**Figure 3:**
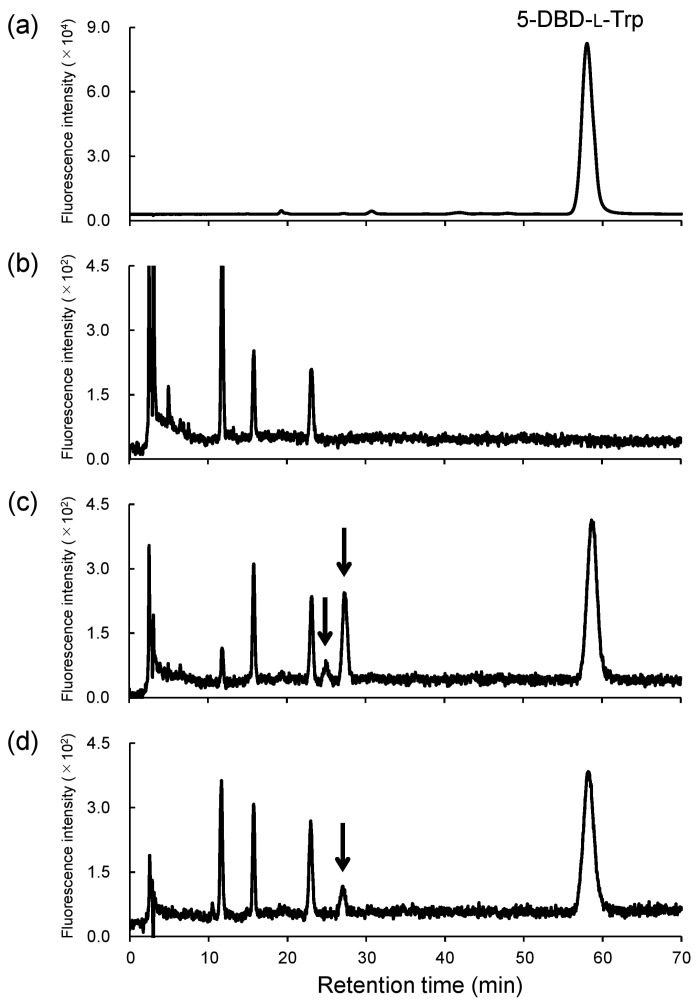
Representative chromatograms of the standard of 5-DBD-l-Trp (**a**) and MD samples at 0–0.5 h (blank sample, (**b**)), 1.5–2.0 h (**c**), and 1.5–2.0 h co-infused with 1-methyl-d-Trp (**d**) obtained from rat kidneys. The arrows in (**c**,**d**) indicate fluorescent peaks originating from metabolites of 5-DBD-l-Trp. l-Trp, tryptophan; DBD, 4-*N*,*N*-dimethylamino-2,1,3-benzoxadiazole; MD, microdialysis.

In the chromatogram obtained by HPLC with fluorescence detection, the prepared 5-DBD-l-Trp was eluted at approximately 58 min (Figure 3a). For the HPLC with fluorescence detection assay of Trp metabolism, the in vivo MD study was carried out using the left kidneys of male Sprague–Dawley rats. In the MD sample, two new fluorescent peaks distinct from 5-DBD-l-Trp were detected at approximately 25 and 27 min during 5-DBD-l-Trp infusion (Figure 3c). The newly detected peaks were deemed to have originated from metabolites of 5-DBD-l-Trp, which might be produced in the kidneys of rats.

#### 2.2.2. LC–MS Analysis

**Figure 4:**
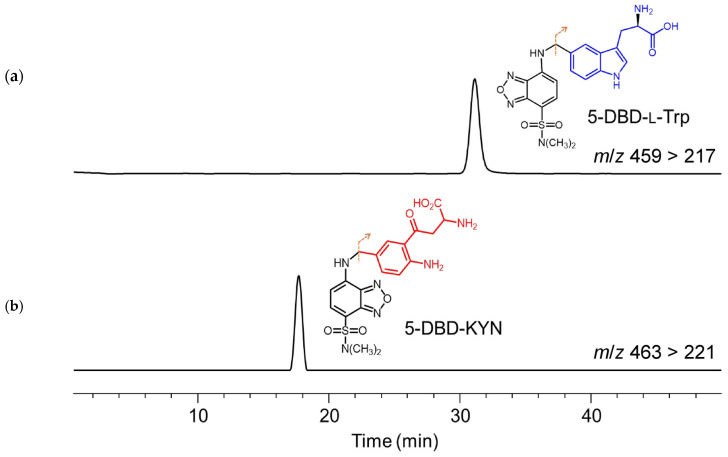
MRM chromatograms of the MD samples from rat kidney infused with 5-DBD-l-Trp, obtained by LC–MS. (**a**) *m*/*z* 459 → 217, (**b**) *m*/*z* 463 → 221. Blue and red colors in (**a**,**b**) are Trp and KYN moieties, respectively. MRM, multiple reaction monitoring; l-Trp, tryptophan; DBD, 4-*N*,*N*-dimethylamino-2,1,3-benzoxadiazole.

LC–MS was used to confirm the presence of 5-DBD-KYN produced from 5-DBD-l-Trp in the MD samples obtained from rat kidneys. The eluted peak at 17–18 min exhibited a mass-to-charge ratio (*m*/*z*) of 463 → 221. Based on the *m*/*z* and the fragmentation ions, it was assumed that the peak was the KYN with DBD structure (Figure 4). This result suggested that 5-DBD-KYN was generated in the MD sample from rat kidney infused with 5-DBD-l-Trp. The other small fluorescent peak (~25 min) could not be identified in this study because it was below the LC–MS/MS limit of detection.

#### 2.2.3. Co-Infusion with 1-Methyl-d-Trp

**Figure 5:**
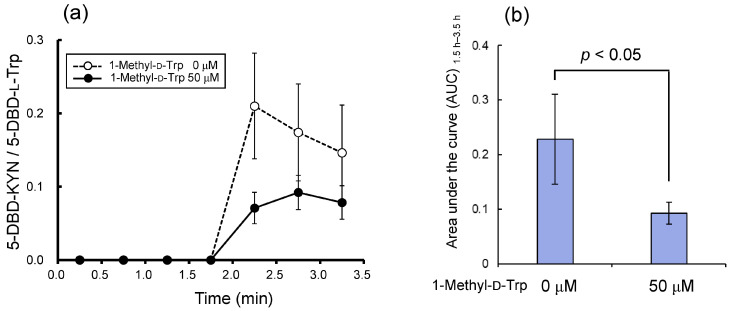
Time-course profiles of the ratio of 5-DBD-KYN to 5-DBD-l-Trp in rat microdialysis (MD) samples from kidney (**a**), and the area under the curve (AUC) values of the ratio of 5-DBD-KYN to 5-DBD-l-Trp (1.5–3.5 h) in rat MD samples co-infused with (*n* = 5) or without (*n* = 4) 50 µM 1-methyl-d-Trp (**b**). l-Trp, tryptophan; DBD, 4-*N*,*N*-dimethylamino-2,1,3-benzoxadiazole.

When an IDO inhibitor, 1-methyl-d-Trp (50.0 μM) [20], was co-infused with 5-DBD-l-Trp, the peak area of the fluorescent 5-DBD-KYN significantly decreased (Figure 5a,b), indicating that IDO may mediate the production of 5-DBD-KYN from 5-DBD-l-Trp. Hence, it is plausible that changes in IDO activity in rat kidney may be assessed by constant infusion of 5-DBD-l-Trp.

## 3. Discussion

In this study, the newly designed and prepared 5-DBD-l-Trp functioned as an analytical tool for fluorometrically monitoring tissue Trp-catabolizing activity, using a rat MD study in the kidney.

DBD is a benzofurazan-type fluorophore. Previously, the fluorescence intensities of benzofurazan-type fluorophores were known to be attenuated by the presence of the indole ring of Trp. In a previous study, the fluorescence peak of Trp tagged with 4-nitrobenzofurazan (NBD) was not detected [21]. Therefore, to determine Trp fluorometrically with HPLC via benzofurazan-type fluorophores such as NBD, on-line electrolytic cleavage of the indole ring in Trp must be performed before fluorescence detection [21,22].

In this study, however, the fluorescence emitted from DBD was observed in the case of 5-DBD-l-Trp. Additionally, the indole ring was cleaved by IDO or TDO (Scheme 1); therefore, the absence of the indole ring may have increased the fluorescence intensity of the DBD moiety. Thus, the in vivo metabolites of 5-DBD-l-Trp could be fluorometrically detected along with 5-DBD-l-Trp using HPLC-fluorescence detection.

Although DBD was attached at the 5-position of the indole Trp ring, our findings suggest that 5-DBD-l-Trp was metabolized to 5-DBD-KYN by one of the Trp-catabolizing enzymes, presumably IDO, in the kidney, because the peak at 27 min decreased and the peak at 25 min completely disappeared (Figure 3) when co-infused with 1-methyl-d-Trp, an IDO inhibitor.

Of the two metabolite peaks, the larger (major) peak likely corresponds to 5-DBD-KYN (RT ≈ 27 min). The smaller peak (RT ≈ 25 min) may represent a downstream metabolite of 5-DBD-KYN; its earlier elution on the reverse-phase column suggests increased hydrophilicity relative to 5-DBD-KYN. Unfortunately, this minor peak could not be identified because it was below the LC-MS/MS limit of identification.

Therefore, IDO activity in the kidney was clearly assessed by monitoring the ratio of the peak area of 5-DBD-KYN to the peak area of 5-DBD-l-Trp in the chromatogram.

In this study, 1-methyl-d-Trp was selected as an inhibitor of IDO because it has already proceeded to clinical trials as a drug for combination therapy with antitumor agents [23,24]. The inhibitory activity of 1-methyl-d-Trp is reportedly weaker than that of 1-methyl-l-Trp [25]. However, we recently observed that chiral inversion of 1-methyl-d-Trp to 1-methyl-l-Trp occurred in rats [26]. Taken together, both 1-methyl-d-Trp and 1-methyl-l-Trp may contribute to attenuating IDO activity in in vivo rat MD experiments.

This study had a limitation. IDO1 and IDO2 isoforms have been identified in mammalian tissues [27]. In this study, it is still unclear which IDO isoform, IDO1 or IDO2, contributed to the metabolism of 5-DBD-l-Trp. To clarify this issue, it will be necessary to use selective inhibitors for IDO1 or IDO2. We intend to conduct this research after the development of such inhibitors.

In conclusion, this study demonstrated that it is possible to utilize 5-DBD-l-Trp as a probe for Trp metabolism research by applying the 5-DBD-l-Trp to in vivo experiments. This approach to designing fluorophore-attached Trp derivatives, combined with tissue MD studies and HPLC analysis, will be useful for evaluating IDO activity in tissues in vivo. In future studies, the present Trp derivative, 5-DBD-l-Trp, will be applied to studies on Trp metabolism using cell culture or clinical samples related to psychiatric disorders and cancer.

## 4. Materials and Methods

### 4.1. Chemicals

Diethyl ether, ethyl acetate, NaHCO_3_, anhydrous Na_2_SO_4_, trifluoroacetic acid, methanol, 35% HCl, KOH, ethanol (99.5), cobalt (II) chloride hexahydrate, acetic acid, LC–MS-grade CH_3_OH, HPLC-grade formic acid, and ketamine hydrochloride were purchased from FUJIFILM Wako Pure Chemical (Osaka, Japan). Triethylamine, 4-(*N*,*N*-dimethylaminosulfonyl)-7-fluoro-2,1,3-benzoxadiazole (DBD-F), di-*tert*-butyl decarbonate (Boc_2_O), dl-serine, 4-dimethylaminopyridine (DMAP), and acetic anhydride were purchased from Tokyo Chemical Industry (Tokyo, Japan). Acylase I from *Aspergillus melleus* and (1*H*-indol-5-yl)methanamine, ammonium formate (HCO_2_NH_4_), 1-methyl-d-Trp, and xylazine hydrochloride were purchased from Sigma Aldrich Co. LLC (Saint Louis, MO, USA).

Sterilized Ringer’s solution was purchased from Otsuka Pharmaceutical Factory, Inc. (Tokushima, Japan).

### 4.2. Preparation of 5-DBD-l-Trp Derivative

#### 4.2.1. Tert-butyl ((1H-indol-5-yl)methyl)carbamate (**2**)

A primary amine of (1H-indol-5-yl)methanamine (**1**) (10 mmol) in ethanol (20 mL) was reacted with Boc_2_O (15 mmol) in the presence of triethylamine (20 mmol) for 30 min at room temperature. The reaction mixture was evaporated under reduced pressure, and the residues were extracted with ethyl acetate (100 mL). The extracts were washed with saturated NaHCO_3_ aqueous solution (50 mL), and the organic layer was then dried over anhydrous Na_2_SO_4_. The organic solvent was evaporated under reduced pressure, and the residue was purified using flash chromatography (SEPAFLASH^®^, Santai Science Inc., Quebec, Canada, 21.3 × 172.7 mm, 40–63 μm) and eluted with a mixed organic solvent, *n*-hexane/ethyl acetate (9:1). The fraction containing compound (**2**) was isolated and evaporated under reduced pressure to obtain a white solid (yield 70.0%).

*m*/*z* 247.1361 (calculated for M+H 247.1368), ^1^H-NMR (400 MHz, CHLOROFORM-D) δ 8.70 (s, 1H, indoleNH), 7.48 (s, 1H, ArH), 7.24 (d, *J* = 8.2 Hz, 1H, ArH), 7.09 (t, *J* = 2.7 Hz, 1H, ArH), 7.04 (d, *J* = 8.2 Hz, 1H, ArH), 6.44 (t, *J* = 2.1 Hz, 1H, ArH), 4.96 (s, 1H, CONH), 4.36 (d, *J* = 5.3 Hz, 2H, CH_2_), 1.48 (d, *J* = 8.2 Hz, 9H, tert-BuH). ^13^C-NMR (101 MHz, chloroform-D) δ 155.9, 135.1, 129.6, 127.7, 124.8, 121.6, 119.4, 111.2, 101.9, 79.2, 45.1, 28.3.

#### 4.2.2. 2-Acetamido-3-(5-(((tert-butoxycarbonyl)amino)methyl)-1H-indol-3-yl)propanoic acid (**3**)

Compound (**2**) (7.01 mmol) was mixed with dl-serine (14.0 mmol) in the presence of DMAP. Subsequently, acetic acid (50 mL) and acetic anhydride (5.0 mL) were added, and the mixture was heated at 80 °C for 60 min. The reaction mixture was poured into EtOAc (50 mL) in a separatory funnel and extracted with saturated Na_2_CO_3_ aq. solution (100 mL), followed by extraction three times with 5% KOH (50 mL). The aqueous phase was combined, acidified with 35% HCl, and extracted twice with EtOAc (50 mL). The organic layer was dried with anhydrous Na_2_SO_4_ and evaporated in vacuo. The residue was purified by flash chromatography Ultrapack ODS-SM-50B^®^ (Yamazen Corp., Osaka, Japan), and the fraction containing (**3**) was isolated and evaporated under reduced pressure to obtain a slightly brown solid (**3**) (yield 55.9%).

*m*/*z* 376.1864 (calculated for M+H 376.1872), ^1^H-NMR (400 MHz, DMSO-D6) δ 10.70 (d, *J* = 1.8 Hz, 1H, indoleNH), 8.06 (d, *J* = 7.8 Hz, 1H, NH), 7.39 (s, 1H, NH), 7.25 (d, *J* = 8.5 Hz, 1H, ArH), 7.15 (t, *J* = 6.0 Hz, 1H, ArH), 7.10 (d, *J* = 2.1Hz, 1H, ArH), 6.99 (dd, *J* = 8.5, 1.4 Hz, 1H, ArH), 4.46 (td, *J* = 8.2, 4.9 Hz, 1H, α-CH), 4.20 (d, *J* = 6.2 Hz, 2H, CH_2_), 3.19−3.13 (m, 1H, β-CH_2_), 2.98 (dd, *J* = 14.7, 8.9 Hz, 1H, β-CH_2_), 1.81 (s, 3H, AcH), 1.40 (s, 9H, tert-BuH). ^13^C-NMR (101 MHz, DMSO-D6) δ 173.6, 169.3, 155.7, 135.2, 129.9, 127.1, 123.8, 120.8, 116.6, 111.1, 109.8, 77.5, 53.0, 44.2, 28.3, 27.1, 22.4.

#### 4.2.3. 2-Acetamido-3-(5-(((7-(*N*,*N*-dimethylsulfamoyl)benzo[c][1,2,5]oxadiazol-4-yl)amino)methyl)-1H-indol-3-yl)propanoic acid (**4**)

Compound (**3**) (3.92 mmol) was added and stirred with trifluoroacetic acid (10 mL) for 30 min at room temperature. After evaporation, 2% methanol in diethyl ether (100 mL) was added to the residue (pinkish oil), and the supernatant was removed. The precipitate was dried under reduced pressure. The dried precipitate (0.83 mmol) was dissolved in ethanol (99.5%, 5.0 mL), and DBD-F (1.1 equivalent) and triethylamine (0.5 mL) were added and stirred for 120 min at room temperature. Subsequently, the reaction mixture was poured into a separatory funnel, and liquid–liquid extraction between EtOAc (100 mL) and saturated NaHCO_3_ aqueous solution (50 mL) was carried out. H_2_O (70 mL) was added to the aqueous phase, which was acidified with 35% HCl at approximately pH 1 and subsequently extracted with EtOAc (50 mL). The organic phase was dried over anhydrous Na_2_SO_4_. The organic layer was evaporated in vacuo, and the residue was purified using flash chromatography with Ultrapack ODS-SM-50B^®^ (Yamazen Corp. 26 × 300 mm, 50 μm), eluted with a mixed polar solvent, H_2_O–methanol (1:1) at 25 mL/min. The fraction containing compound (**4**) was isolated and evaporated in vacuo to obtain a yellow-orange solid (**4**) (yield 24.0%).

*m*/*z* 523.1216 (calculated for M+Na 523.1370), ^1^H-NMR (400 MHz, methanol-D4) δ 7.68 (d, *J* = 8.1 Hz, 1H, ArH), 7.57 (s, 1H, ArH), 7.25 (d, *J* = 8.4 Hz, 1H, ArH), 7.10–7.05 (m, 2H, ArH), 6.16 (d, *J* = 8.2 Hz, 1H, ArH), 4.67 (dd, *J* = 8.0, 5.2 Hz,1H, α-CH), 4.58 (s, 2H, indole-5-CH_2_), 3.27–3.25 (m, overwrapped with the solvent signal), 3.07 (dd, *J* = 14.8, 8.1 Hz, 1H, β-CH_2_), 2.65 (d, *J* = 9.3 Hz, 6H, N(CH_3_)_2_), 1.86–1.81 (m, 3H, acetylH). ^13^C-NMR (101 MHz, methanol-D4) δ 175.2, 173.1, 147.8, 145.8, 142.7, 141.5, 137.5, 129.0, 128.6, 125.2, 122.2, 118.5, 112.7, 111.1, 107.5, 100.4, 54.7, 38.2, 28.5, 22.5, 22.5, 22.4

#### 4.2.4. 2-Amino-3-(5-(((7-(*N*,*N*-dimethylsulfamoyl)benzo[c][1,2,5]oxadiazol-4-yl)amino)methyl)-1H-indol-3-yl)propanoic acid (5-DBD-l-Trp) (**5**)

Compound (**4**) (0.237 mmol) was suspended in 50 mM phosphate buffer (pH 7.5) (25 mL) and reacted with acylase I (Sigma Aldrich) in the presence of cobalt (II) chloride for 39 h at 45 °C. The reaction mixture was loaded onto a flash chromatography system, Ultrapack ODS-SM-50B^®^ (Yamazen Corp., 26 × 300 mm, 50 μm), and eluted with a mixed solvent, acetic acid–H_2_O–methanol (0.1:50:50), at a flow rate of 20 mL/min. The fraction containing 5-DBD-l-Trp was isolated and evaporated in vacuo to obtain a yellow-orange solid (5-DBD-l-Trp) (yield 41.8%).

*m*/*z* 459.1283 (calculated for M+H 459.1372), ^1^H-NMR (400 MHz, DMSO-D6) δ 10.92 (s, 1H, indole NH), 9.03 (s, 1H, CH_2_NH), 7.75 (d, *J* = 8.1 Hz, 1H, ArH), 7.69 (s, 1H, ArH), 7.30 (d, *J* = 8.4 Hz, 1H, ArH), 7.19 (d, *J* = 1.5 Hz, 1H, ArH), 7.15 (d, *J* = 8.4 Hz, 1H, ArH), 6.35 (d, *J* = 8.1 Hz, 1H, ArH), 4.64 (s, 2H, CH_2_NH), 3.44 (dd, *J* = 9.0**, 3.8** Hz, 1H, α-CH_2_), 3.30 (dd, *J* = 15.1*, 3.5** Hz, 1H, β-CH_2_), 2.94 (dd, *J* = 15.1*, 9.3** Hz, 1H, β-CH_2_), 2.64 (s, 6H, N(CH_3_)_2_). ^13^C-NMR (101 MHz, DMSO-D6) δ 169.8, 146.4, 144.4, 141.0, 140.5, 135.7, 127.4, 127.2, 124.7, 120.8, 117.9, 111.5, 109.6, 105.1, 99.5, 54.7, 47.0, 37.4, 27.1.

### 4.3. Measurement of the Fluorescence Spectrum

A solution of 5-DBD-l-Trp was prepared in the HPLC mobile phase described in Section 4.5, namely a mixed solution (A:B = 4:1) of 20 mM ammonium formate in H_2_O/MeOH (9/1, *v*/*v*) (A) and MeOH (B). The excitation and fluorescence spectra of 5-DBD-l-Trp (10 µM) were measured using a spectrofluorometer, F-7000 (Hitachi High-Tech Corporation, Tokyo, Japan). For comparison, a solution of l-Trp (1.0 µM) was also measured under the same conditions.

### 4.4. MD Experiments

Male Sprague–Dawley rats (7 weeks old) were purchased from the Jackson Laboratory (Kanagawa, Japan) and were used after housing for at least 1 week in a temperature- and humidity-controlled experimental animal facility at Toho University. The rat MD study was carried out according to our previous study [12]. Briefly, the rats were anesthetized with ketamine/xylazine (Fujifilm-Wako Pure Chemical Corporation, Osaka, Japan, 90/10 mg/kg/mL in saline) and were fixed in a supine position on a heating pad (Bio Research Center Co., Ltd., Tokyo, Japan). After making an incision in the abdomen, a linear MD probe (10 mm membrane; BASi, West Lafayette, IN, USA) was implanted into the left kidney, with the dialysis membrane of the MD probe completely embedded in the kidney. The probe position was secured using a hemostatic matrix, Integran^®^ (Nippon Zoki Pharmaceutical Co., Ltd., Osaka, Japan) and surgical glue, Aron Alpha^®^ (Daiichi-Sankyo Co., Ltd., Tokyo, Japan). One end of the linear probe was connected to a 1 mL gastight syringe, and Ringer’s solution was perfused at a rate of 1.0 μL/min via a syringe pump (11 plus; Harvard apparatus, Holliston, MA, USA) for 2 h.

Subsequently, 5.0 μM 5-DBD-l-Trp dissolved in phosphate-buffered Ringer’s solution was infused into the kidney at 1.0 μL/min (*n* = 4) for 1.5 h. For co-infusion with an IDO inhibitor, 1-methyl-d-Trp, the inhibitor was dissolved at 50 μM in the Ringer’s solution throughout the MD experiment (*n* = 5). The MD samples obtained from the dialysis membrane were collected every 30 min on ice. An aliquot of 10 μL of each MD sample was diluted five times with the HPLC mobile phase (A), and the resultant solution (10 μL) was subsequently analyzed using HPLC with fluorescence detection.

### 4.5. HPLC with Fluorescence Detection

The HPLC system comprised an autosampler (AS-4050i), an intelligent pump (PU-4180), a column oven (CO-2065 plus), an interface box (LC-Net II/ADC), and a fluorescence detector (FP-4025; Jasco Corp., Tokyo, Japan). The fluorescence detection wavelength was set at 560 nm with an excitation wavelength of 450 nm, based on previous studies using the DBD moiety as a fluorophore [28,29]. The temperature of the autosampler tray was set at 4 °C. The analytical column was an Inert Sustain^®^ column (250 × 4.6 mm; i.d., 5 μm, GL Sciences Inc., Tokyo, Japan) maintained at 40 °C in the column oven. The mobile phase, consisting of 20 mM ammonium formate in H_2_O/MeOH (9/1, *v*/*v*) (A) and MeOH (B), was pumped at a constant flow rate of 1.0 mL/min using the following elution program: 0–70 min, B% = 20; 70.01–80 min, B% = 100; and 80.01–110 min, B% = 20. The injection volume was 10 μL. The obtained chromatograms were analyzed using ChromNAV^®^ ver. 2 software (Jasco Corp.).

### 4.6. LC-MS

A triple quadrupole LCMS-8040 mass spectrometer (Shimadzu, Kyoto, Japan) attached to an electrospray ionization interface was used for LC-MS/MS. Two pumps (LC–20AD), an autosampler (SIL-20AC), a column oven (CTO-20A), and PC software (LabSolutions ver. 5.113, Shimadzu) were used. The temperature of the autosampler tray was set at 4 °C. The analytical column was an Inert Sustain^®^ column (250 × 2.1 mm; i.d., 3 μm, GL Sciences Inc., Tokyo, Japan) maintained at 50 °C in the column oven. The mobile phase, consisting of 20 mM ammonium formate in H_2_O/MeOH (9/1, *v*/*v*) (A) and MeOH (B), was pumped constantly at a flow rate of 0.2 mL/min using the following elution program: 0–50 min, B% = 20; 50.01–60 min, B% = 100; 60.01–70 min, B% = 20. The injection volume was 5.0 μL. The desolvation line and heatblock temperatures were adjusted to 250 and 400 °C, respectively. The flow rates of the nebulizing and drying gases, ion-spray voltage, and collision-induced dissociation gas pressure were 3.0 and 10 L/min, 4.5 kV, and 230 kPa, respectively.

The fragmentation behavior of 5-DBD-l-Trp was optimized by LC–MS/MS using a 1.0 mM standard solution (exact mass: 458.1372). At a collision energy (CE) of –15 V, the precursor ion (*m*/z 459) was fragmented at the orange-dotted position in Figure 4 to produce a fragment ion at *m*/*z* 217. Based on these results, the presumed metabolite, 5-DBD-KYN (exact mass: 462.1321), was able to provide the same fragmentation pattern, yielding a product ion at *m*/*z* 221. Thus, the multiple reaction monitoring (MRM) conditions were established as follows: precursor ions at *m*/*z* 459 and 463 and product ions at *m*/*z* 217 and 221 for 5-DBD-l-Trp and 5-DBD-KYN, respectively, both at a CE of –15 V.

Thus, ions were detected in MRM mode (*m*/*z* 459 > 217 for DBD-l-Trp and *m*/*z* 463 > 221 for the metabolite, 5-DBD-KYN).

### 4.7. Data Analysis

Data are expressed as the mean ± standard error. To compare the area under the curve between the two groups (with and without inhibitor), a *t*-test was performed using BellCurve for Excel (Social Survey Research Information Co., Ltd., Tokyo, Japan).

## Data Availability

The original contributions presented in this study are included in the article. Further inquiries can be directed to the corresponding author.

## Abbreviations

The following abbreviations are used in this manuscript:

Trp	tryptophan
HPLC	high-performance liquid chromatography
DBD	4-*N*,*N*-dimethylamino-2,1,3-benzoxadiazole
LC-MS	liquid chromatography–mass spectrometry
